# Safety of Teduglutide for Managing Patients with Short Bowel Syndrome: A Systematic Review and Meta-analysis

**DOI:** 10.12669/pjms.41.9.12604

**Published:** 2025-09

**Authors:** Shuai Liu, Xiaowei Yu, Feifei Ye, Liangxian Jiang

**Affiliations:** 1 Shuai Liu Department of Gastrointestinal Surgery, Taizhou Hospital of Zhejiang Province, Taizhou, Zhejiang Province 317000, P.R. China; 2 Xiaowei Yu Department of Gynecology, Taizhou Hospital of Zhejiang Province, Taizhou, Zhejiang Province 317000, P.R. China; 3 Feifei Ye Department of Gastrointestinal Surgery, Taizhou Hospital of Zhejiang Province, Taizhou, Zhejiang Province 317000, P.R. China; 4 Liangxian Jiang Department of Gastrointestinal Surgery, Taizhou Hospital of Zhejiang Province, Taizhou, Zhejiang Province 317000, P.R. China

**Keywords:** Adverse Reactions, Meta-Analysis, Short Bowel Syndrome, Teduglutide

## Abstract

**Objectives:**

Short bowel syndrome (SBS), characterized by insufficient absorptive surface after extensive intestinal resection, results in chronic intestinal failure and reliance on parenteral nutrition. This meta-analysis aimed to examine the adverse events associated with teduglutide treatment for SBS.

**Methodology:**

Comprehensive search was conducted in PubMed, Embase, Cochrane Central Register of Controlled Trials (CENTRAL) and CINAHL databases for studies of SBS patients who received teduglutide as part of their therapeutic regimen and time period of search was from inception of database till last date of search (30 June 2023). Given nature of research question and absence of control arms, we performed a single-arm meta-analysis to pool prevalence estimates of each adverse event using random-effects model. Heterogeneity was assessed by chi-square test and I^2^ statistic and publication bias was evaluated using Egger’s test.

**Results:**

Overall, 17 studies were included in analysis. Meta-analysis revealed that gastrointestinal side effects were prevalent in about 49% of patients [95% CI: 0.32-0.66]. Upper respiratory tract infections, central venous catheter-related infections, pyrexia and sepsis were seen in 29% [95% CI: 0.20-0.39], 11% [95% CI: 0.04-0.20], 23% [95% CI: 0.11-0.37] and 22% [95% CI: 0.13-0.31] of patients, respectively. Hepatic, biliary and pancreatic complications, electrolyte imbalances, dehydration, cardiac complications, injection site reactions and urinary tract infections had prevalence of 11% [95% CI: 0.07-0.17], 13% [95% CI: 0.00-0.37], 11% [95% CI: 0.08-0.16], 5% [95% CI: 0.00-0.12], 13% [95% CI: 0.05-0.24] and 12% [95% CI: 0.06-0.21], respectively.

**Conclusion:**

This meta-analysis highlights the significant adverse event profile associated with teduglutide in the management of SBS. While the drug presents an essential therapeutic option, healthcare professionals must carefully monitor the occurrence of these side effects.

**Meta-analysis: PROSPERO:**

CRD42023447985

## INTRODUCTION

Short Bowel Syndrome (SBS) is a complex and challenging clinical condition that is defined as a significant loss of functional small intestine, whether due to surgical resection or congenital defect, leading to severe malabsorption of nutrients.^1^ SBS patients require long-term parenteral nutrition (PN) or intravenous (IV) hydration.^2^ The symptomology of SBS is ranging from diarrhoea, malnutrition and weight loss, to more complex manifestations such as liver disease, renal impairment and life-threatening sepsis.^3^ SBS is associated with a considerable burden, not only in terms of morbidity and mortality, but also in terms of the profound impact on the quality of life of patients.^4^

Management strategies for SBS are multidimensional and should aim to optimize intestinal adaptation, reduce PN dependency and improve overall patient survival and quality of life.^2^ Pharmacological agents are considered the most effective option due to their potential to enhance residual intestinal function. The recombinant glucagon-like peptide-2 (GLP-2) analogue teduglutide is one such novel therapy for SBS. Studies show that teduglutide effectively promotes mucosal growth and enhances absorptive capacity.^5^

Teduglutide’s role in managing SBS has gained considerable attention since its approval by the U.S. Food and Drug Administration (FDA) in 2012 and the European Medicines Agency (EMA) in 2013.^6^ Several studies have showed that teduglutide treatment may reduce parenteral nutrition/intravenous (PN/IV) volume, with some patients even achieving complete independence from parenteral support.^7^-^10^ However, studies show that teduglutide use may be associated with side effects, such as potential risks of neoplastic growth due to its proliferative effects on the gut mucosa, the likelihood of intestinal obstruction and the reported cases of biliary and pancreatic disorders.^11^

There is still no consensus on the safety of teduglutide. While some studies (including previous review)^11^ report a higher incidence of adverse events associated with the treatment, others argue these adverse events may be inherent to SBS’s nature and not necessarily a consequence of teduglutide therapy.^7^-^10^

This review aimed to summarize the existing data and establish a safety profile of teduglutide in managing SBS patients. To clarify our research question, we have applied the PEO format as follows:

- Population (P): Adults diagnosed with SBS receiving teduglutide
- Exposure (E): Administration of teduglutide
- Outcome (O): Prevalence and spectrum of treatment-emergent adverse events, including gastrointestinal symptoms (e.g., abdominal pain, nausea, vomiting), infections, hepatobiliary complications, electrolyte disturbances, dehydration, cardiac events and injection-site reactions.

## METHODOLOGY

This review was performed in adherence to the Preferred Reporting Items for Systematic Reviews and Meta-Analyses (PRISMA) 2020 guidelines.^12^

### Eligibility Criteria:

This study focused on analysing the safety of teduglutide in managing patients with SBS. The review encompassed studies with patients who were diagnosed with SBS and received teduglutide as part of their therapeutic regimen. We imposed no limitations on age, gender, ethnicity, or geographic location.

### Intervention group:

SBS patients who were administered teduglutide.

### Outcomes

The primary outcomes analysed were the prevalence of specific adverse events following teduglutide administration, such as gastrointestinal side effects, upper respiratory infections (URI), pyrexia, central venous catheter (CVC) related infections, urinary tract infections (UTIs), hepatobiliary complications, cardiac complications, sepsis, dehydration, electrolyte imbalances and injection-related reactions. We included randomized controlled trials (RCTs), observational studies, cohort studies, between the inception of searchable databases and June 2023. The last date of search was 30 June 2023. To mitigate publication bias, we considered both published and grey literature.

We conducted a systematic search of electronic databases, including PubMed, Embase, Cochrane Central Register of Controlled Trials (CENTRAL) and CINAHL. Reference lists of included studies and relevant reviews were searched manually for additional studies. The authors were contacted for additional data or clarification of study details when necessary. We combined terms associated with “Short Bowel Syndrome,” “Teduglutide,” and the specific “adverse events” mentioned above, using both Medical Subject Headings (MeSH) and relevant keywords.

### Study Records:

### Data Management:

Retrieved studies were managed using EndNote X9 citation management software. We eliminated duplicates and the remaining articles were screened for inclusion.

### Selection Process:

Two independent reviewers (SL and XY) performed the screening of titles and abstracts of the retrieved studies. Full texts of potentially eligible studies were then evaluated for inclusion. Any disagreements were resolved through discussion or by consulting with a third reviewer (FY).

### Data Collection Process:

Two reviewers (SL and XY) independently extracted data using a standardized Form. The extracted data included study characteristics (authors, publication year, study design, setting), participant characteristics (number of participants, age, gender, severity of SBS), details of teduglutide administration and the reported adverse events. We also collected information on funding sources and potential conflicts of interest.

### Risk of Bias in Individual Studies:

To evaluate the risk of bias, the Cochrane Risk of Bias Tool,^13^ and Newcastle Ottawa Scale 14 were used for RCTs and for observational studies, respectively. The assessments were independently performed by two reviewers (FY & LJ), with disagreements resolved through discussion or by consulting a third reviewer (SL).

### Data Synthesis:

Meta-analysis was performed when studies were sufficiently homogeneous in terms of design, participants, interventions and outcomes. Given the nature of research question and absence of control arms, we performed a single-arm meta-analysis to pool prevalence estimates of each adverse event. We used a random-effects model to account for potential heterogeneity among studies and double arcsine transformation to stabilize the variance.^15^ Measures of effect included pooled prevalence for each adverse event. Forest plots visually represented the individual study effects and the pooled prevalence, with point estimates of effects in individual studies (represented by squares proportional to study weight) and the associated 95% confidence intervals (CI) (represented by horizontal lines). A diamond shape at the bottom of each forest plot signified the overall prevalence and its CI. We assessed heterogeneity using the I^2^ statistic. 15 We used funnel plots and Egger’s regression test to evaluate publication bias and checked for selective reporting within studies by comparing reported outcomes with those listed in study protocols or trial registries. Sensitivity analysis was performed to check the robustness of the effect estimate.

## RESULTS

**Fig.1:**
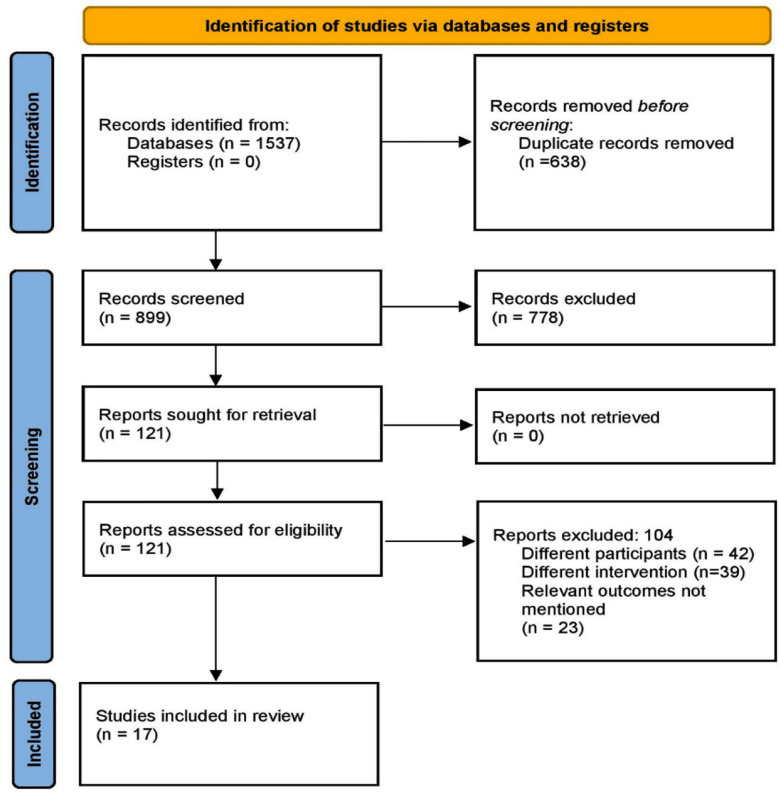
Search strategy.

Initial search across the databases identified 1537 citations. Following duplicates removal, 121 full-text articles were retrieved. These studies underwent secondary screening and a total of 17 studies were included as satisfying the eligibility criteria. Fig.1.^6^-^10^,^16^-^28^

he included studies were published between 2011 to 2023 and conducted in various countries including Japan, Latin America, North America, Europe and others. The study designs were equally diverse, including RCTs, retrospective studies and prospective studies. The duration of teduglutide treatment varied from 42 days to 22 months. The dose of teduglutide ranged from 0.020 mg/kg/day to 0.1 mg/kg/day. Both genders were represented, though not equally in all studies (Table I). Most studies (9 out of 17) had higher risk of bias (Table-IIA & B).

**Table-I:** Included studies.

Study	Location	Study design	Sample size	Duration of treatment	Dose of Teduglutide (mg/kg/day)	Mean age (in years)	Gender distribution Male: Female (%)	Risk of bias
Busoni 2021	Latin America	Retrospective	3	22 months	0.05	9.7	100%:0%	Low
Carter 2017	USA & United Kingdom	Prospective	42	12 weeks	.0.05 for 15 0.025 for 14 0.125 for 8 participants	3	67%:33%	High
Chiba 2023	Japan	RCT	24	24 weeks	0.05	7	79.2%:20.8%	Low
Hill 2019	NA	Prospective	16	6 months	0.05	NR	NR	High
Jeppesen 2011	Multinational	RCT	67	24 weeks	0.1	48.6	44.8%:55.2%	Low
Jeppesen 2013	Multinational	RCT	68	24 weeks	0.05	49.4	43.8%:56.2%	High
Kinberg 2021	USA	Retrospective	8	10 months	0.05	5	50%:50%	High
Kocoshis 2016	24 centers in North America and Europe	Prospective	59	24 weeks	0.05 for 26 0.025 for 24 participants	7	70%:30%	High
Lambe 2021	France	Prospective	25	48 weeks	0.05	10	NR	Low
Martinez 2019	Argentina	Retrospective	4	42 weeks	0.05	12	75%:25%	High
Mercer 2019	NA	Prospective	55	6 months	0.05	NR	NR	Low
Nakamura 2023	Japan	RCT	18	24 weeks	0.05	40.9	72.2%:27.8%	Low
O’Keefe 2013	Multinational	Prospective	52	52 weeks	0.1	48.1	53.8%:46.2%	Low
Ramos-Boluda 2020	Spain	Prospective	17	12 months	0.05	68 months	NR	High
Ribeiro-Mourao 2021	Portugal	Prospective	4	6 months	0.05	9	25%:75%	Low
Schwartz 2016	9 European and North American countries	Prospective	88	24 weeks	0.05	50.9	46.6%:53.4%	High
Sigalet 2015	Canada	Prospective	7	42 days	0.020	5.4 months	NR	High

NA-Not applicable; NR-Not reported; RCT-randomized controlled trial; USA - United States of America.

**Table-IIA:** Risk of bias assessment of RCTs.

Study	Randomization	Deviation from intended intervention	Missing outcome data	Measurement of outcome	Selection of Reported Results	Risk of bias
Chiba 2023	Low	Low	Low	Low	Low	Low
Jeppesen 2011	Low	Low	Low	Low	Low	Low
Jeppesen 2013	Low	High	Some concerns	High	Some concerns	High
Nakamura 2023	Low	Low	Low	Low	Low	Low

**Table-IIB:** Risk of bias assessment of observational studies.

Study	Selection	Comparability	Outcome	Overall Points	Risk of bias
Busoni 2021	3 points	2 points	2 points	7 points	Low
Carter 2017	1 point	0 point	1 point	2 points	High
Hill 2019	1 point	1 point	1 point	3 points	High
Kinberg 2021	1 point	0 point	0 point	1 point	High
Kocoshis 2016	1 point	0 point	1 point	2 points	High
Lambe 2021	4 points	2 points	2 points	7 points	Low
Martinez 2019	1 point	0 point	0 point	1 point	High
Mercer 2019	4 points	2 points	2 points	8 points	Low
O’Keefe 2013	3 points	2 points	2 points	7 points	Low
Ramos-Boluda 2020	1 point	0 point	1 point	2 points	High
Ribeiro-Mourao 2021	3 points	2 points	2 points	7 points	Low
Schwartz 2016	1 point	0 point	1 point	2 points	High
Sigalet 2015	1 point	1 point	1 point	3 points	High

### Gastrointestinal adverse events:

**Fig.2:**
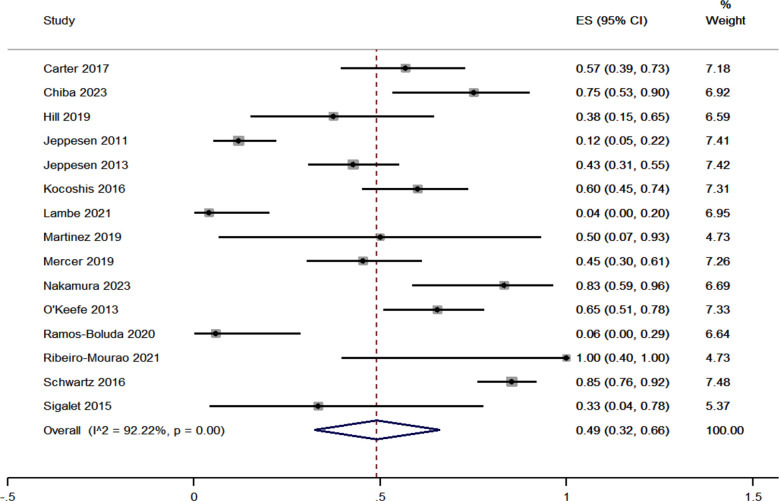
Forest plot showing the pooled estimate for gastrointestinal adverse events in patients receiving teduglutide for short bowel syndrome.

The meta-analysis of gastrointestinal adverse events associated with teduglutide treatment for SBS involved 15 studies. Pooled estimate of effect size (ES) was 0.49 [95%CI, 0.32 to 0.66]. Fig.2 The heterogeneity chi-square p-value was less than 0.01, indicating significant heterogeneity amongst the studies. The variation in ES attributable to heterogeneity (I^2^ statistic) was 92.22%. Egger’s test had non-significant p-value (p=0.75), indicating the possibility of the lack of publication bias. Sensitivity analysis did not alter the magnitude of effect estimate. Meta-regression was performed using the potential covariates such as study design, quality, setting, mean age and sample size. The overall multivariable meta-regression model was able to explain 42% of the between-study variance.

### Upper Respiratory Infections:

**Fig.3:**
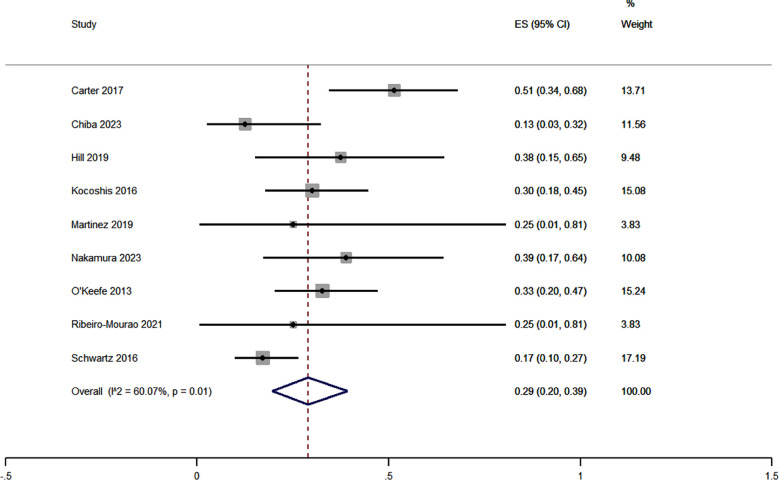
Forest plot showing the pooled estimate for upper respiratory tract infections in patients receiving teduglutide for short bowel syndrome.

The meta-analysis of upper respiratory infections in SBS patients who were treated with teduglutide incorporated data from nine studies. Pooled ES was 0.29 [95% CI, 0.20 to 0.39] Fig.3. The heterogeneity chi-square value was 20.03 (p=0.01), suggesting a significant level of heterogeneity among the studies. The I^2^ statistic was calculated as 60.07%. Sensitivity analysis did not alter the magnitude of effect estimate.

### CVC related infections:

**Fig.4:**
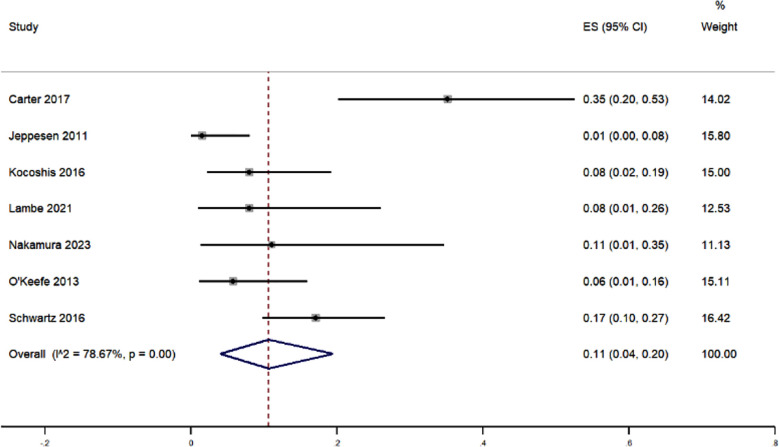
Forest plot showing the pooled estimate for central venous catheter related adverse events in patients receiving teduglutide for short bowel syndrome.

The pooled results from the meta-analysis of CVC related infections as an adverse event in patients treated with teduglutide showed that, on average (pooled prevalence) 11% of patients experienced this complication, with the 95% CI ranging from 4% to 20%. Fig.4 The heterogeneity chi-square test resulted in a value of 28.13 (p<0.01). I^2^ statistic of 78.67% also indicated a substantial degree of heterogeneity. Sensitivity analysis did not alter the magnitude of effect estimate. Meta-regression was performed using the potential covariates such as study design, quality, setting, mean age and sample size. The overall multivariable meta-regression model was able to explain only 11% of the between-study variance.

### Pyrexia:

**Fig.5:**
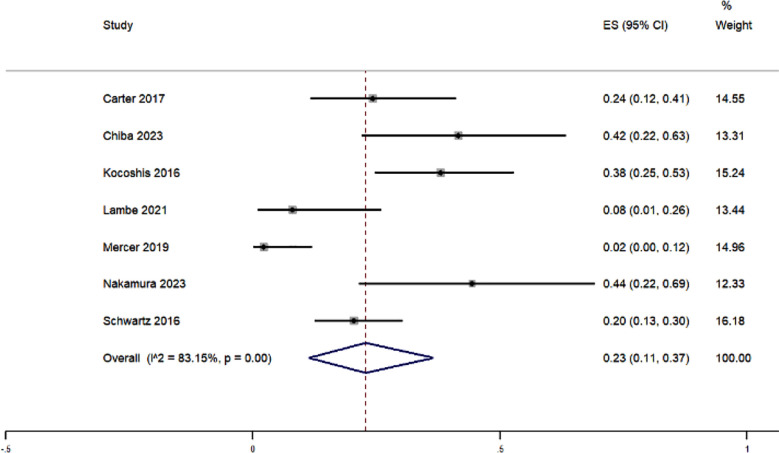
Forest plot showing the pooled estimate for pyrexia in patients receiving teduglutide for short bowel syndrome.

The pooled results from the meta-analysis of pyrexia (fever) as an adverse event in patients treated with teduglutide showed that, on average, 23% of patients experienced this complication, with the 95% CI ranging from 11% to 37%. Fig.5 This analysis incorporated data from seven studies and had heterogeneity among studies (I^2^ statistic=83.15%). Sensitivity analysis did not alter the magnitude of effect estimate. Meta-regression was performed using the potential covariates such as study design, quality, setting, mean age and sample size. The overall multivariable meta-regression model was able to explain 23.58% of the between-study variance.

### Sepsis:

The meta-analysis results of sepsis as an adverse event in patients treated with teduglutide indicated that approximately 22% of patients experienced this serious complication, with a 95% CI ranging from 13% to 31%. heterogeneity among studies was low, as indicated by an I^2^ statistic of 16.99% and a chi-square test yielding a p-value of 0.30. Sensitivity analysis did not alter the magnitude of effect estimate.

### Hepatic, biliary and pancreatic complications:

Our meta-analysis of hepatic, biliary and pancreatic complications as adverse events in patients treated with teduglutide indicated that an estimated 11% of patients experienced these complications, with a 95% CI ranging from 7% to 17%. There was no evidence of significant heterogeneity among the studies (I^2^=0%) and chi-square test resulted in a p-value of 0.66. Sensitivity analysis did not alter the magnitude of effect estimate.

### Electrolyte imbalances:

Our meta-analysis for electrolyte imbalances as adverse events in patients treated with teduglutide showed a pooled estimate of 13%. The 95% CI for this estimate ranged widely from 0% to 37%. There was significant heterogeneity among the included studies, as illustrated by the I^2^ statistic of approximately 79% and a chi-square test resulting in a p-value of 0.01. Sensitivity analysis did not alter the magnitude of effect estimate.

### Dehydration:

The meta-analysis for dehydration as an adverse event in patients treated with teduglutide demonstrated a pooled estimate of 11%. The 95% CI for this estimate ranged from 8% to 16%. There was no substantial heterogeneity, as indicated by I^2^= 0%, Chi-square test with a p-value of 0.53. Sensitivity analysis did not alter the magnitude of effect estimate.

### Cardiac complications:

The meta-analysis for cardiac-related adverse events in patients treated with teduglutide showed a pooled estimate of 5%. The 95% CI for this estimate ranged from 0% to 12%. The heterogeneity between the studies was somewhat notable, as the I^2^ statistic was 51.21%. The Chi-square test for heterogeneity had a p-value of 0.13, which is not statistically significant at the conventional 0.05 level. Sensitivity analysis did not alter the magnitude of effect estimate.

### Injection related reactions:

The meta-analysis for injection site-related adverse events in patients treated with teduglutide showed a pooled estimate of 13%. The 95% CI for this estimate ranged from 5% to 24%. The heterogeneity between the studies was high, as the I^2^ statistic was 71.03%. Chi-square test for heterogeneity had a p-value of <0.001. Sensitivity analysis did not alter the magnitude of effect estimate.

### Urinary tract infections:

The meta-analysis for urinary tract infections in patients treated with teduglutide showed a pooled estimate of 12%, with 95%CI of 6% to 21%. There was a moderate heterogeneity between the studies, (I^2^ = 43.04%). Chi-square test for heterogeneity had a p-value of 0.15. Sensitivity analysis did not alter the magnitude of effect estimate.

## DISCUSSION

The present meta-analysis offers a detailed investigation into the adverse event profile of teduglutide and unequivocally affirm that teduglutide treatment is associated with a range of adverse events in SBS patients. However, the probability of such adverse events varies substantially, as does the heterogeneity among the included studies we analysed. This is a critical observation that underscores the importance of considering the individual study data and specific patient characteristics when viewing our results.

Our results showed that 49% of SBS patients who received teduglutide reported gastrointestinal adverse events. This is a major complication in patients who are already dealing with an underlying gastrointestinal condition like SBS.^29^ However, our findings need to be interpreted with caution due to the high level of heterogeneity among the studies. The significant heterogeneity raises the question of the potential influence of various confounders, including disease severity, concurrent medications and patient-specific factors, which might not have been adequately controlled for in the individual studies.^30^

It is paramount to consider the inherent nature of SBS when discussing gastrointestinal adverse events. Patients with SBS are predisposed to a range of gastrointestinal issues due to their underlying condition and this could potentially confound the association with teduglutide treatment. The prevalence of gastrointestinal complications in this patient population is inherently high and discerning the extent to which teduglutide contributes to these adverse events is complex. Further research is needed to untangle these associations and ascertain the specific role of teduglutide in exacerbating gastrointestinal complications in SBS patients.

URIs, CVC-related infections and pyrexia emerged as common adverse events as well, with estimated prevalence of 29%, 11% and 23%, respectively. Our results suggest that teduglutide may have potential immunomodulatory effects due to its action on the GLP-2 receptor which is known to influence immune function.^31^

Similarly, sepsis, a serious and potentially fatal complication, was observed in 22% of patients. While the underlying reasons for the increased risk of sepsis are not entirely clear from our analysis, they could be related to a complex interplay of factors, including teduglutide’s potential immunomodulatory effects, the increased infection risk associated with central venous catheters and the generally compromised health status of SBS patients.^32^

It is critical to note that SBS patients are generally at an elevated risk for infections due to their compromised nutritional status, frequent need for central venous catheters and potential for intestinal dysbiosis. This heightened risk for infections could contribute to the prevalence of adverse events such as upper respiratory tract infections, CVC-related infections, pyrexia and sepsis observed in our study. While teduglutide’s immunomodulatory effects could play a role, it is important to distinguish the baseline risk of infections in this vulnerable population from the potential additional risk imposed by the medication. Future studies should aim to dissect these complex relationships to provide clearer guidance on the management of infectious complications in SBS patients on teduglutide.

Our analysis showed that hepatic, biliary and pancreatic complications occurred in approximately 11% of SBS patients who received teduglutide. Since teduglutide may promote intestinal and pancreatic growth, it is plausible that these effects might extend to other components of the digestive system, leading to the observed adverse events.

We showed that 13% and 11% of patients experienced electrolyte imbalances and dehydration, respectively. While these findings might be reflective of the baseline disease status in SBS patients, they further emphasize that the management of SBS patients should involve a careful balance between medication use, dietary modifications and supportive treatments to optimize electrolyte balance and hydration status.

Cardiac complications and urinary tract infections were found to be relatively less common, affecting approximately 5% and 12% of SBS patients, respectively. However, given the potential severity of these complications, healthcare providers should remain vigilant for any signs suggestive of these conditions in patients on teduglutide. Interestingly, injection site reactions, a relatively minor but not insignificant concern, were observed in about 13% of patients. While generally manageable, these reactions could influence medication adherence and hence, need to be proactively addressed.

Our results further strengthen the notion that the management of SBS patients should go beyond merely prescribing medication and should include regular monitoring and early intervention for the adverse events while maintaining a personalized patient-centric approach. Given the infectious complications and other adverse events observed, it is crucial to underscore the importance of vigilant monitoring and proactive management of SBS patients on teduglutide. The safety concerns should be weighed against the potential benefits of the medication, taking into account the patient’s baseline risk for complications due to their underlying condition. A nuanced understanding of the safety profile of teduglutide in the context of SBS is indispensable for making informed clinical decisions and ensuring optimal patient outcomes.

In light of the diverse patient population affected by SBS, it is imperative to consider the potential regional differences in the manifestation and prevalence of side-effects associated with teduglutide and other GLP-2 analogues. Patients from different world regions may exhibit varying responses to these medications due to a multitude of factors, including genetic predispositions, environmental influences, dietary habits and access to healthcare resources.

Specifically focusing on the Asian demographic, there is a paucity of comprehensive data delineating the safety profile of teduglutide in this population. This gap in knowledge necessitates cautious interpretation of the findings from predominantly Western-centric studies when applying them to Asian patients. Moreover, the potential for unique side-effects or variations in their prevalence cannot be overlooked. For instance, the dietary patterns and microbiome compositions, which are significantly influenced by geographical and cultural factors, could play a pivotal role in modulating the patients’ responses to GLP-2 analogue treatment.

Given the inclusion of various study designs in our meta-analysis, it is imperative to discuss the adverse events in the context of both the interventions and the underlying disease of SBS. The nature of SBS, coupled with its associated complications, necessitates a nuanced approach in evaluating the causality of reported adverse events. In our analysis, all adverse events were meticulously listed and examined. However, we acknowledge the complexity in distinguishing the effects solely attributed to the drug from those stemming from the disease itself.

### Limitations:

Firstly, there is a significant heterogeneity observed across several of the adverse event categories that may be due to varying patient populations, disease severities and concurrent medication use. The inclusion of a wide array of study designs, while broadening the scope of our analysis, also introduces variability that must be considered when interpreting our findings. The complexity of SBS as a disease further complicates the evaluation of causality, blurring the lines between drug-related adverse events and those arising from the underlying condition. We have endeavored to address these challenges through a comprehensive and meticulous analysis, providing clear distinctions where possible and openly discussing the inherent complexities of this research area. Our study is also limited by the quality of the included studies, with over half having high risk of bias. This limitation could potentially impact the validity of our findings and thus, should be kept in mind when interpreting the results. In the present analysis, a notable limitation is the absence of data from novel GLP-2 analogues, which are currently under investigation. The unavailability of this data is a gap in the literature that we acknowledge and it is factor that readers should consider when interpreting our findings. This limitation underscores the importance of continuous updates and re-evaluations of the safety profiles of GLP-2 analogues as new evidence emerges. Some included studies (for example, Martinez 2019)^28^ were available only as abstracts or conference proceedings, which often lack full methodological details and complete outcome data, thereby increasing the risk of incomplete reporting bias.

Despite these limitations, this meta-analysis provides a comprehensive overview of the adverse events associated with teduglutide treatment in SBS patients. It adds valuable evidence to the existing body of literature and aids in refining the risk-benefit considerations for the use of teduglutide in clinical practice. The findings of our study should be seen as a steppingstone towards more personalized patient care in SBS, guiding clinicians in shared decision-making processes and management strategies.

Although our analysis focused on teduglutide, it is important to consider these findings in the context of the broader class of GLP-2 analogues. All agents share agonism at the GLP-2 receptor, promoting intestinal mucosal growth and fluid absorption, which may underlie common adverse events such as abdominal discomfort, fluid overload and electrolyte imbalances.^31^ Differences in molecular structure, half-life and dosing frequency (e.g., glepaglutide and apraglutide in development) could modulate the incidence or severity of these events, but no data yet suggest a fundamentally distinct safety profile. Clinicians should therefore anticipate a similar spectrum of AEs with next-generation GLP-2 analogues, while remaining alert for compound-specific signals as larger trials and post-marketing surveillance emerge.

As the field of therapeutics for Short Bowel Syndrome continues to evolve, we acknowledge the impending arrival of data from ongoing studies on novel GLP-2 analogues. Recognizing the potential of these upcoming interventions to further our understanding of the safety profiles and efficacy of GLP-2 analogues, we anticipate incorporating their findings into future iterations of our analysis. This future work will aim to compare the substances in depth, thereby shedding light on whether the observed side effects are class-effects or specific to individual substances. Our established methodological framework and expertise in this area ensure that we are well-prepared to integrate and analyze new data as soon as they become available, contributing timely and valuable insights to the community.

## CONCLUSION

This meta-analysis has showed that amongst adult patients with short bowel syndrome treated with teduglutide, gastrointestinal adverse events (most commonly abdominal pain, nausea and vomiting) and injection-site reactions occur at pooled prevalences of approximately 30–40% and 15–20%, respectively. These findings underscore that teduglutide has a generally favourable safety profile but requires routine monitoring for these treatment-emergent events.

### Authors’ contributions:

**SL, LJ:** Literature search, Study design and manuscript writing.

**XY, FY and SL:** Data collection, data analysis and interpretation. Critical Review

**SL, LJ:** Manuscript revision and validation and is responsible for the integrity of the study.

All authors have read and approved the final manuscript.
